# Regulatory T Cells: Angels or Demons in the Pathophysiology of Sepsis?

**DOI:** 10.3389/fimmu.2022.829210

**Published:** 2022-02-25

**Authors:** Yu-lei Gao, Ying Yao, Xiang Zhang, Fang Chen, Xiang-long Meng, Xin-sen Chen, Chao-lan Wang, Yan-cun Liu, Xin Tian, Song-tao Shou, Yan-fen Chai

**Affiliations:** ^1^ Department of Emergency Medicine, Tianjin Medical University General Hospital, Tianjin, China; ^2^ Department of Emergency Medicine, Rizhao People’s Hospital of Shandong Province, Rizhao, China; ^3^ Department of Medical Research, Beijing Qiansong Technology Development Company, Beijing, China

**Keywords:** sepsis, regulatory T cells, pathophysiology, checkpoints, secondary infections

## Abstract

Sepsis is a syndrome characterized by life-threatening organ dysfunction caused by the dysregulated host response to an infection. Sepsis, especially septic shock and multiple organ dysfunction is a medical emergency associated with high morbidity, high mortality, and prolonged after-effects. Over the past 20 years, regulatory T cells (Tregs) have been a key topic of focus in all stages of sepsis research. Tregs play a controversial role in sepsis based on their heterogeneous characteristics, complex organ/tissue-specific patterns in the host, the multi-dimensional heterogeneous syndrome of sepsis, the different types of pathogenic microbiology, and even different types of laboratory research models and clinical research methods. In the context of sepsis, Tregs may be considered both angels and demons. We propose that the symptoms and signs of sepsis can be attenuated by regulating Tregs. This review summarizes the controversial roles and Treg checkpoints in sepsis.

## Introduction

A study of the global burden of disease from 1990 to 2017 showed that an estimated 48.9 million (38.9-62.9) sepsis cases were recorded globally and 11.0 million (10.1-12.0) sepsis-related deaths were reported in 2017, representing 19.7% (18.2-21.4) of global deaths (1). In China, one-fifth of patients admitted to intensive care units (ICU) have sepsis and their 90-day mortality rate is 35.5%. It is estimated that the annual medical costs of the 230,000 septic patients admitted to China’s ICUs are about US $4.6 billion, which is a huge medical and social burden (2). In 2015, over 1.9 million deaths occurred in 605 disease-surveillance points in mainland China, and the standardized sepsis-related mortality incidence was 66.7 deaths per 100,000 population (3). Despite the 37% (11.8-54.5) decrease in age-standardized sepsis and the 58% (47.7-57.5) decrease in mortality from 1990 to 2017, aggressive infection source control, early appropriate antibiotic treatment, titration, compression therapy, and improved organ support measures, sepsis remains one of the major causes of global mortality (1–7).

Regulatory T cells (Tregs) are a subset of CD4+ T lymphocytes with negative immunomodulatory functions. They maintain peripheral immune tolerance to control immune responses to prevent exaggerated responses to infections and harmless antigens, and prevent autoimmunity. Due to the extensive regulatory role that Tregs play in the immune system, they have considerable potential as treatment for various diseases (8–10). The discovery that forkhead box P3 (Foxp3) is a key transcription factor in the differentiation and function of Tregs has multiple implications for understanding how the immune system functions and for developing therapeutic interventions for autoimmune diseases, infectious diseases, and malignancies (11–13).

**Figure 1:**
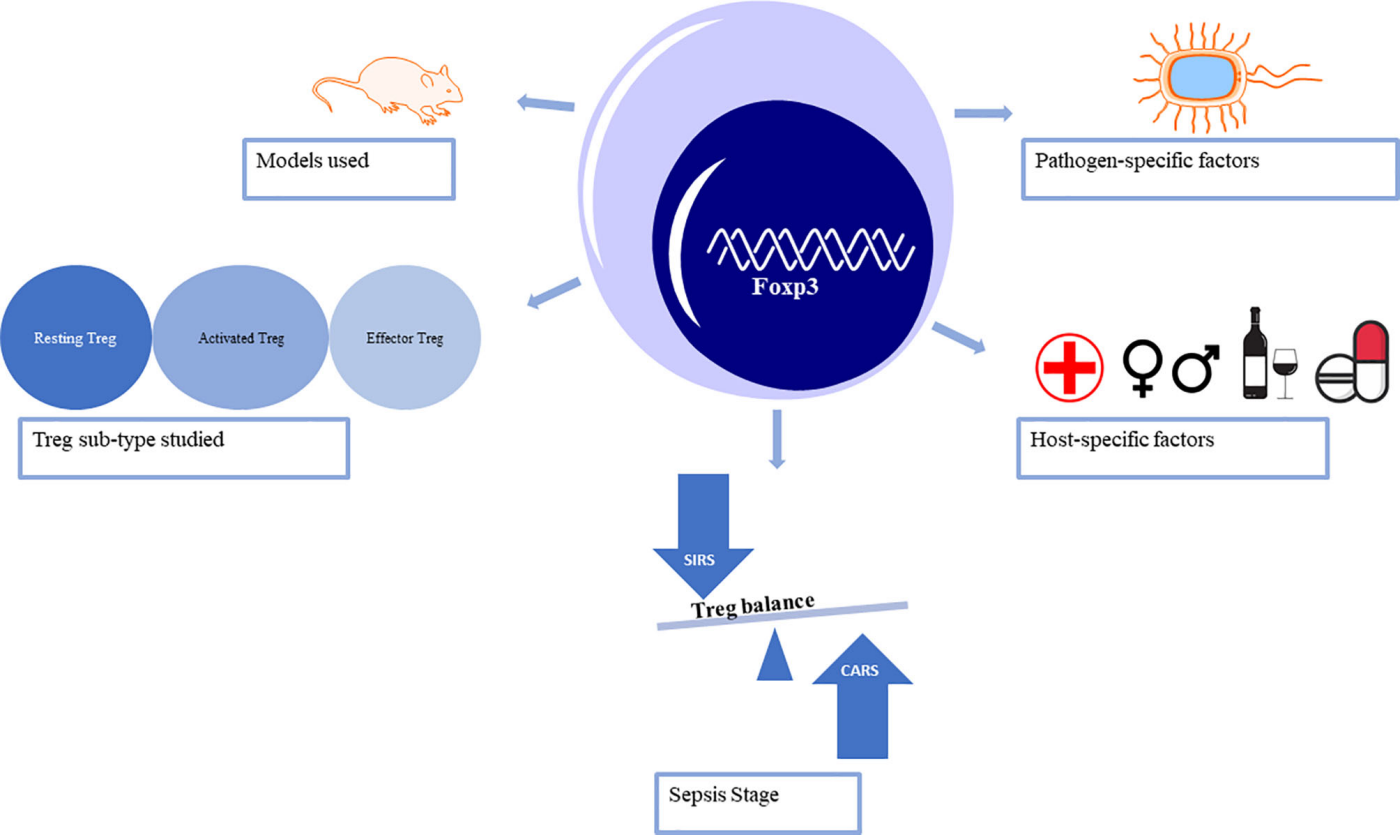
Factors that lead to the controversy surrounding the role of regulatory T cells in sepsis.

Over the past two decades, Tregs have been a focus in sepsis-induced immune-inflammatory dysfunction research and the hotspot strategy in immunotherapy and checkpoint inhibition (14, 15). In sepsis, reduced T cell function is associated with increased expression of Foxp3 (16–18). CD4+ T helper 17 cells (Th17) represent the pro-inflammatory subpopulation, while Tregs promote anti-inflammatory effects (19–22). This review explores the current controversial, and sometimes conflicting, conclusions about Tregs in the pathophysiology of sepsis. Both laboratory and clinical research methods and models need to be considered to begin to understand the precise role of Tregs throughout the stages of sepsis (**Figure 1**).

## Sepsis

Research on the epidemiology, prevention, and management of sepsis is an important topic for critical care medicine, surgery, and anesthesiology, where clarification of the complex pathophysiological mechanism of sepsis is a fundamental problem (14, 23, 24). Since 1992, the definitions of sepsis, severe sepsis, and septic shock, as well as associated clinical and laboratory studies have relied on the presence of infection and the characteristics of systemic inflammatory response syndrome (SIRS) (1, 24–28). However, efforts to inhibit this hyper-inflammatory response syndrome by blocking pro-inflammatory cytokines, such as interleukin (IL)-1β and tumor necrosis factor (TNF)-α, ultimately fail to yield survival benefits (26, 27). Physicians have emphasized the evaluation of sepsis-induced organ dysfunction when they conduct the diagnosis and treatment of sepsis, especially based on the sequential (sepsis-related) organ failure assessment (SOFA), national early warning score and modified early warning score, rather than quick SOFA (qSOFA) (4–6, 24, 28–32).

**Figure 2:**
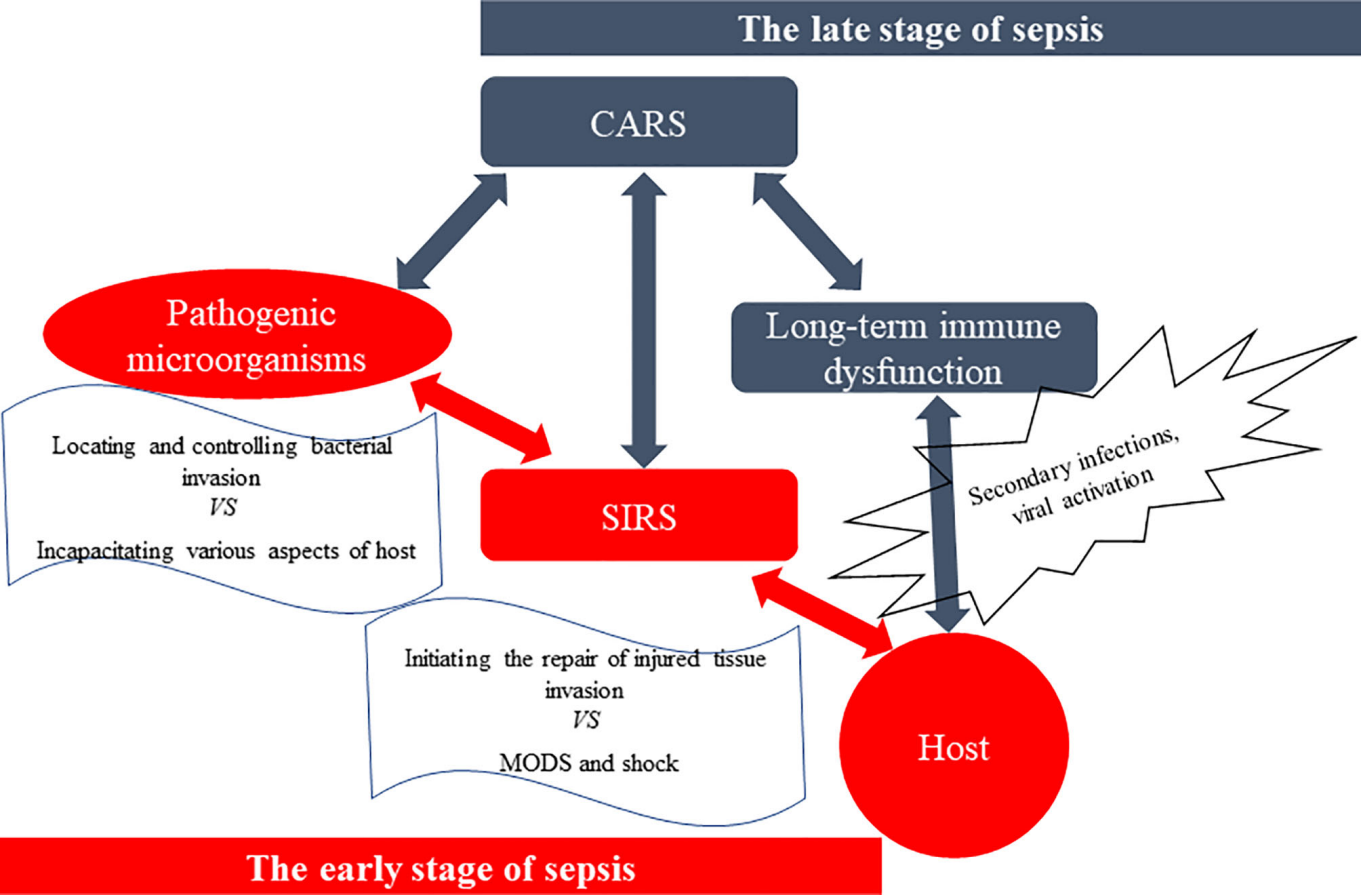
The antagonism between the host and pathogenic microorganisms. The spread of pathogens, especially Gram-negative bacteria, and their products [such as lipopolysaccharide (LPS), etc.] causes systemic inflammatory response syndrome (SIRS), which leads to multiple organ dysfunction syndromes (MODS) and shock, and even death. However, surviving patients suffer from a stage of compensatory anti-inflammatory response syndrome (CARS), especially immunosuppression, and experience a long-term immune dysfunction called immuno-paralysis. And they are more susceptible to secondary infections, increased viral activation, and reduced 5-year survival rate, compared to those who do not have sepsis.

Antagonism between the host and pathogenic microorganisms is a complex pathophysiological reaction: pathogens seek an advantage by incapacitating various aspects of host defenses while the host seeks to control the bacterial invasion and initiate repair of injured tissues (33, 34) (**Figure 2**). Compared with Sepsis 3.0 criteria, the definition of Sepsis 2.0 and 1.0, as well as guidelines for the diagnosis and treatment of sepsis, focus more on pathophysiological mechanisms (4, 25, 26, 29, 35). The first three days of sepsis are defined as the early stage; in these cases, more than 80% of patients first seek emergency medicine according to their clinical manifestations of the biological systemic immune-inflammatory response (26, 36–43). As sepsis management techniques continue to improve, most patients survive the SIRS-induced “cytokine storm” in the early stage of sepsis and begin the late stage dominated by compensatory anti-inflammatory response syndrome (CARS) (44–46). Compelling experimental and clinical evidence has indicated that SIRS and CARS occur early and simultaneously in sepsis, and immunosuppression may persist for months or even longer from the onset of sepsis (4, 15, 26, 45–51). Importantly, immunosuppression is the cause of such aggravation, which increases the chance of secondary infections and viral activation. This complicates multiple organ dysfunction syndromes (MODS), extends hospital length of stay, and may even leads to death (15, 45, 46, 49).

Approximately 60-70% of septic deaths occur in the late stage (≥3 days) and most deaths were associated with ICU-acquired complications, including nosocomial infections (52). T cell apoptosis and dysfunction contributes to sepsis-induced immunosuppression (15, 26, 46, 53, 54). Intervention strategies, such as anti-programmed cell death (PD)-1/PD-L1 mAb, blocking cytotoxic T lymphocyte antigen (CTLA)-4, and blocking 2B4, have improved survival in experimental models of sepsis and recent clinical trials through improved T cell-induced immunosuppression (46, 55–58). The degree of sepsis-induced inflammation, including the level of immunosuppression, is defined by specific host factors (such as age, gender, alcoholism, repeated nosocomial infection, frequency in hospital, chronic comorbidities, immunosuppressant use, malignant tumor, site of infection, splenectomy, trauma, and stress state) (59–66), pathogen status (such as multiple drug-resistant organisms, malaria, SARS-CoV-2) (7, 26, 67, 68), and the duration of sepsis (6, 28, 35, 44, 46, 61, 69, 70).

## Treg Heterogeneity in Sepsis

Combined single-cell, TCR, and other analyses of Tregs and conventional CD4+Foxp3– T cells (Tconv) demonstrate that Tregs are highly heterogeneous cells in homeostasis and disease (71–73). Treg cells can be either thymus-derived or peripherally induced by naive CD+ T cells. Phenotypically, Tregs are identified by markers they possess such as the transcriptional regulator Forkhead box (Foxp3). Based on the expression levels of Foxp3, Tregs can be either resting Tregs with weak inhibitory potential, activated Tregs with strong inhibitory potential, or cytokine secreting non-suppressive Tregs also called effector Tregs (74). Sepsis influences the heterogeneous characteristics of Tregs from the aspects of percentage (22, 74), absolute number (67, 75), phenotypes (15, 47, 50, 58, 75–79), cytokine and chemokine secretion (80–82), and stability (12, 13, 60) (**Tables 1**–**4**). Coordination between the innate and adaptive immune systems plays a crucial role in the host’s responses to infection. Even after sepsis recovery, the mechanism and cellular characteristics of the immune system change due to the different characteristics of host immune and pathogen status (23, 59–65, 84, 105, 120, 121).

**Table 1:** Observational studies using animal models focused on the characteristics of T_regs_ in sepsis.

Observational study	Species	Model	Observation time	Specimen Source	T_regs_	Immunological characteristics	Outcome
He, et al. (23)	Mouse	Recurrent sepsis (Three LPS stimulations, once every 5 ds)	5 ds after the last LPS injection	Spleens Lung	Percentage and absolute number↑	percentage and number of CD4^+^ T cells↓ Percentage of CD8^+^ T cells↓ CD69 and CD28 of CD4^+^ T cells↓ PD-1 and Tim-3 of CD4^+^ T cells↑ MHC-II of antigen-presenting cells↓	Antiviral immune responses (secondary virus infection) ↓
		Acute sepsis (One LPS stimulations)	12, 24, and 48 hs after LPS injection		Percentage↑	Percentage and number of total T cells↓ percentage and number of CD4/8^+^ T cells↓ CD69 at 12hs↑, 24, and 48 hs↓ MHC-II of antigen-presenting cells↑	Antiviral immune responses (secondary virus infection) →
Gaborit, et al. (83)	Mouse	*Pseudomonas aeruginosa* induced “two-hit” model	Day 3 for T cell phenotype Day 4 for lung injury in the double-hit model	Spleens Lung	Activation↑ TNFR2^pos^ Tregs↑ *Gizmo, Mki67* (Ki67), *Irf4*, *Prdm1* (Blimp 1) and *Havcr2* (TIM-3) ↑ CD62, CD25, CTLA-4 and IL-10↑ percentage↑	Number of splenic CD4^+^T cells↓	Susceptibility to secondary pneumonia↑
Saito, et al. (84)	Mouse	CS-induced model SAE	24hs, 6, 8 10, and 30 ds after induced	Brain Blood Spleen and other lymph nodes	Percentage and absolute number (brain)↓	Microglia, neuroinflammation, and neutrophil infiltration (brain)↑ astrocytes (brain)↓ CD4/8^+^ T cell accumulation (brain)↑ T cell (blood and spleen)↓	The brain-blood barrier was disrupted (24hs) Anxiety-like behaviors↑ Marble burying test↑ Open-field test↓ Forced swimming test↓ Depression
George, et al. (17)	Mouse	Acute *DenV* infection and 7 days later followed with l*isteria monocytogenes* induced “two-hit” sepsis	3, 7,10,15 ds after induced	Spleen Blood	Proliferation (number and percentage)↑ GITR, CTLA-4, Foxp3, CD40L, CD44, CD62L, and CD69↑ IL-10, and TGF-β↑	CD4/CD8^+^ T cell responses↓ IFN-γ↓	Susceptibility↑ Attenuates DHF/DSS
Baek, et al. (85)	Preterm pigs	LPS stimulated Staphylococcus epidermidis challenge	6, 12, and 24 hs, day 5, 7, and 9 after stimulated or challenged	Cord blood Blood	Percentage and absolute number↑	Genes related to both innate and adaptive immunity↓ Blood neutrophil and platelet counts↓ Neutrophil phagocytosis capacity↓	Severe septic responses↑ 9-ds incidence and severity of spontaneous infections↑ Overall birth weight↓
Shrestha, et al. (86)	Neonatal mouse	LPS stimulated once daily on postnatal days (PNDs) 3–5	PND7 or PND14	Lung	Percentage and absolute number↓	*CCL2, CCL3, CXCL1, IL-1β*, and *TNF-α*↑ *IL-10*↑	Survival↓ Body weight↓ Alveolar simplification↑ Pulmonary vascular simplification↑ Lung cell proliferation↓, and apoptosis↑
Qiu, et al. (79)	*G protein-coupled receptor 174*- KO Mouse	LPS stimulated CLP	24 hs after stimulated or challenged	Thymus Lung	*Ctla-4, Pdcd-1, and IL-10*↑ CTLA-4 and IL−10↑	M2 macrophages↑ IL-6 and TNF-α↓	2 and 7-ds survival↑ Resistant to inflammatory shock↑ Lung injury↓
Zhou, et al. (87)	PTEN^M−K^ Mouse	LPS-induced ALI	24 hs after induced	Blood Lung BALF	Percentage and absolute number↑ Foxp3 and TGF-β↑	TGF-β↑ Neutrophil accumulation↓ RORγt, IL-17A, TNF-α, and IL-1β↓	6-ds survival↑ Lung injury↓ Weight↑
Andrade, et al. (88)	Mouse	Received LPS injection each day for 5 days, and followed with CLP	4 hs after CLP	Spleen Blood	Absolute number (received LPS injection) ↑ Absolute number (after CLP) ↓	IL-2, IL-6, TGF-β, and INF-γ (received LPS injection)↑ Th17(received LPS injection)↑ Th17(after CLP) ↓	72-hs survival↑
Cao, et al. (89)	TLR4^−/−^ mouse	CLP	24 hs after CLP	Lung Liver Spleen Blood	Percentage and absolute number↓ Apoptosis↓ *Foxp3* and *Tlr4*↓	IL-10 and IL-4↓ IL-2 and TNF-α↑	72-hs survival↑ Lung, liver injury↓
Fay, et al. (78)	CD43^-/-^ mouse	CLP	24 hs after CLP	Spleens	Percentage ↓	Number of CD4^+^ T cells↓ Numbers of CD4/8^+^ central memory and effector memory cells↓ Apoptosis of central memory T cells↑ IL-2-secreting CD4+ T cells↓ IL-4-secreting CD4+ T cells↑ Th17↓	7-ds survival↓
Bomans, et al. (69)	Mouse	CLP	3 months after CLP	Spleens Blood Bone marrow	Percentage (spleens)↑	Enlarged spleens with higher weights↑ CD11b^+^ F4/80^−^ splenic monocytes↑ Ly6C^+^ inflammatory monocytes↑ Ly6C^−^ alternative monocytes↓ CD11b^+^ F4/80^+^ macrophages↑	There is no long-term impact of sepsis on the systemic immune response in mice 12 weeks after CLP.
Ahmadi, et al. (64)	Tumor mouse	Induction of systemic *candidiasis*	8 ds after induction	Blood Spleens Renal Liver	Percentage (spleens and tumor) ↑	ratio of IFN-γ/IL-4↓ IL-10↑	Relative tumor volume↑
Hu, et al. (90)	Mouse	Injected with PC61 before a two-hit model	24 hs two-hit	Lung BLFC Spleens	Percentage and absolute number↓	IL-1β, IL-6, and IL-17A↑ IL-10↓	3 and 7-ds survival↑ Bacterial colonies↓ Lung injury↓

Compared with the control group, “↑” represents up-regulation, increase or enhancement； “↓” represents down-regulation, decrease or inhibition, and “→” represents no change.

**Table 2:** Observational studies using septic patients or combined animal models focused on the characteristics of T_regs_ in sepsis.

Observational study	Species	Model	Observation time	Specimen Source	T_regs_	Immunological characteristics	Outcome
Yin, et al. (74)	Humans	Severe sepsis/septic shock patients with severe neutropenia	Day of PICU admission	Blood	Percentage↑	CRP, PCT, IL-6, IL-10, and IFN-γ↑	28-ds survival↓
Jiang, et al. (76)	Humans	Septic patients	Day of ICU admission	Blood	PD-1↑↑ CD28, PD-L1, and CD86↑	No data	SOFA scores↑ 28-ds survival↓
Youssef, et al. (91)	Neonates	Vitamin D deficiency	After enrolled	Cord blood	Percentage ↓	Total lymphocytes, CD3^+^ T lymphocytes, CD4^+^ T-helper, CD8^+^ T-cytotoxic lymphocytes, and CD4^+^CD45RA^+^ naïve T cells↓	16.27% of infants with a 25-OHD deficiency were admitted with sepsis No cases of sepsis in the normal 25-OHD group
Carvelli, et al. (37)	Humans	Septic shock patients	24 and 72 hs after admission	Blood	Percentage↓	Lymphocytes (CD3^+^ T cells and CD3^−^CD56^+^ NK) ↓ HLA-DR↓ Innate lymphoid cells 1 count↑ Innate lymphoid cells 2 count↓ Innate lymphoid cells 3 count↓ Innate lymphoid cells 3 percentage↑	secondary infections↑
Arens, et al. (92)	Humans	Abdominal sepsis	Over 5 ds	Blood	No distinguishable trends in the percentage	B and NK cell counts↓ IL-8↓ Th17 cells↑	Day 21, 5/26 patients showed no *candida* colonization or invasive candidiasis (IC), 13/26 patients colonization was detected, and 8/26 patients were diagnosed with IC.
Xu, et al. (60)	Humans	Septic patients	Days 1, 3, 5, 7, 10, 14, 21 and 28 after sepsis	Blood	Foxp3 (survivors)↑	HLA‐DRA (survivors)↑ Th1 and Th2 cells(especially non-survivors)↓ Th17 (survivors)↑ T‐bet (Th1) and GATA‐3 (Th2) had a linear correlation with HLA‐DRA 59 survivors, 19 non‐survivors	
Yu, et al. (80)	Humans	Septic patients	After admission	Blood	The ratio of IL-10^+^ Tregs to total Tregs↓	TSLP↑ Number of Th1 cells↑ IL-1β, IL-6, IFN-γ, and TNF-α↑	134 patients had hyperleukocytosis and a high neutrophil ratio Mortality↑ Stays in the intensive care unit↑
Liu, et al. (75)	Humans	Septic patients	Ds 1 and 7	Blood	Day 1, absolute number (non-survivors) ↓ Day 7, percentage and absolute number(non-survivors) ↑ Day 7, percentage↓↓ and absolute number(survivors) ↑↑		BTLA expression on Tregs (non-survivors) ↑↑ Day 1, BTLA on CD4^+^ T cells was in patients with severe sepsis↓ day 7, BTLA on CD4^+^ T−cells in both survivors and non-survivors↑ BTLA/Tregs were positively correlated with SOFA
Greenberg, et al. (93)	Humans	*Staphylococcus aureus*	After positive S. aureus blood culture	Blood	Associated with immunosuppressive medications	Neutrophil-to-lymphocyte count ratio↑ IL-6 and IL-17A↑ Th17↑ Th1↓ Th17 score-to-Th1 score ratios↑	90-ds survival↓
Lu, et al. (40)	Humans	Septic shock	Within 3 ds	Blood	Percentage↑ OX40↑	CD28, CD27, OX40 on CD4^+^ T cells↑ OX40 on CD4^+^CD27^-^CD28^-^ T cells↑ CD4^+^CD27^-^CD28^-^ T cells↓ 4-1BB on CD4/8^+^ T cells↓	28-ds survival↓ SOFA↑
Li, et al. (67)	Humans	Pneumonia induced sepsis	Day of ICU admission	Blood	Percentage and absolute number↓	Th17/Treg, Th1/Th2, and M1/M2 cell radios↑ IL-6, TNF-α, IL-1β, and IL-18↑ HMGB1, RAGE, and IL-17A↑	No data
	SD rats	Pneumonia-derived sepsis rat was induced with *Klebsiella pneumonia*	12 hs after induced	Blood Lung			
Willers, et al. (94)	Infants (newborns and during infancy)	Follow-up observation was conducted for 2 years	First-year of life (ds 1, 3, 10, 30, 90, 180, and 360)	Stool samples Intestine biopsies	A low level of S100 proteins in infants’ fecal samples associated with the development of sepsis and obesity by age 2 years.		
	Newborn S100a9^–/–^mouse	Infected with *Staphylococcus aureus*	24hs after infection	Blood Intestine Mesenterial Celiac lymph nodes Colon contents	Percentage and absolute number (brain)↓	CX3CR1 protein, and *Il10* and *Tgfb1* mRNAs↓	fatal sepsis↑

Compared with the control group, “↑” represents up-regulation, increase or enhancement； “↓” represents down-regulation, decrease or inhibition.

**Table 3:** Intervention studies using animal models focused on the target of T_regs_ in sepsis.

Intervention study	Species	Model	Intervention	Intervention time	Observation time	Specimen Source	T_regs_	Immunological characteristics	Outcome
Sun, et al. (57)	Mouse	Memory mouse (56 ds antigen-experienced) and CLP	TIGIT (αTIGIT Ab)	12 and 24 hs after CLP	48 hs after CLP	Spleens Blood	Activation↓ Differentiation↓ Helios↓ CTLA-4↓ IL-10↓	Apoptosis of memory T cells↑ T cell function↓ IL-10, IL-6,and MCP-1↓	7-ds survival↓
Sun, et al. (95)			CD28 (agonistic anti-CD28 Ab)	Immediately after CLP and at ds 2, 4, and 6 post-CLP	24 hs after CLP		Proliferation and activation↑ IL-10↑ CD127, CD69, Helios and CTLA-4↑	Apoptosis of CD44^hi^ memory CD4^+^ T cells↓ IL-10↑	7-ds survival↑
Tran, et al. (70)	Mouse	Injected with LPS, and 24 hs latter CLP induced “Two-Hit” Model	Bilirubin	Immediately after CLP	24 hs after CLP	Lung Blood	Percentage↑	TNF-α, IL-6, and IFN-γ↓ IL-10, and TGF-β↑ T cell activation↓ IFN-γ-producing cells↓	14-ds survival↑ Lung injury↓
Ge, et al. (96)	Mouse	CLP	IL-38 (rmIL-38)	2 hs before or after severe CLP	48 hs after CLP	Spleens	Immunosuppressive activity↑ IL-10 and TGF-β1↑ Foxp3 and CTLA-4↑	Th2 response (IL-4)↑ Proliferative ability of T cells↓	7-ds survival↑
Zhao, et al. (81)	Mouse	CLP	IL-3 (siRNA, IL-3 Ab)	2, 6, and 12 hs after CLP	48 hs after CLP	Spleens, lung, and liver	Foxp3, CTLA-4, IL-10, and TGF-β↑ Suppressive activity↑ Percentage↑	Hyper-inflammatory response (TNF-α and IFN-γ)↓ Anti-inflammatory response (IL-10)↑	5-ds survival↑ Lung and liver injury↓
Kulkarni, et al. (97)	Mouse	Stool suspension	IL-7	Daily from day 5–9	3.5 months after sepsis	Spleen	Percentage and absolute number (within 1 week after sepsis) ↑	IL-10^+^ B cells (Bregs)↑ CD3^+^CD4^-^CD8^-^ T cells↑ IFN-γ and IL-10↑	No data
Nadeem, et al. (98)	Mouse	LPS-induced ALI	ITK (inhibitor)	Once 30 min before and then 3 times after LPS administration at 12 hourly intervals	48 hs after LPS	BAL	Percentage↑	Total leukocytic and neutrophilic numbers↓ %IL-17A ^+^ CD4^+^ T cells↓	Lung injury↓
Zou, et al. (19)	SD rats	CLP	miR-126 (mimic)	Immediately after CLP	48 hs after CLP	Blood	Percentage and absolute number↑	TNF-α, IL-6, and IL-17↓ IL-10↑ lymphocyte of apoptosis↓ Number of Th17↓	No data
Xia, et al. (20)	Mouse	CLP	Maresin1	1 h after CLP	24 hs after CLP	Lung BALF	Percentage and absolute number↑	IL-1β, TNF-α, IL-6, and IL-17↓ IL-10 and TGF-β↑ Th17/Tregs↓	7-ds survival↑ Lung injury↓
Li, et al. (99)	Mouse	CLP-induced ALI	Excretory secretory products of *Trichinella* *spiralis* adult worms	Immediately after CLP	12 hs after CLP	Lung Blood	Percentage↑	IL-10 and TGF-β↑ TNF-α, IL-6, IL-1β↓ HMGB1, TLR2, and MyD88↓	3-ds survival↑ Lung injury↓
Liu, et al. (21)	Mouse	CLP-induced pancreatic injury	Baicalin	Immediately after CLP	72 hs after CLP	Blood Spleen Pancreatic tissue	Percentage and absolute number↑ Foxp3 (pancreatic tissue)↑	Th1 and Th17 cells↓ T bet and RORγt (pancreatic tissue)↓ IFN-γ and IL-17↓ IL-10↑	Pancreatic injury↓
Liu, et al. (100)	Rats	Burning model	Rhubarb	Immediately after model	12, 24, and 72 hs after CLP	Liver Blood	Percentage and absolute number↑	CD4^+^T cell percentage↓ CD8^+^ T cell percentage↑ CD19^+^B cell percentage↓ NK cell percentage↑	No data
Saito, et al. (59)	Young mouse	day 0, 4, 7, and 10 to inject CS	IL-15	Day 3, 7 and 10	Within 50 days	Blood Spleens Peritoneal lavage fluids	Percentage and absolute number↓	Naïve CD4^+^ T cell↑ PD-1^+^CD4^+^ T cells↓ CD8^+^ T cell↑	Prevent the initial reduction of body weight (Day 3) ↑ Survival ↑
	Aged mouse						Percentage and absolute number↓	CD4^+^ T cells↑ Naïve CD4^+^ T cell↑ PD-1^+^CD4^+^ T cells↓ Naïve CD8^+^ T cells↑ PD-1^+^CD8^+^ T cells↓	Survival ↑
Ge, et al. (101)	Mouse	CLP	IL-36 (IL-36β)	2 hs before or after CLP	48 hs after CLP	Spleens	Tregs were required IL-10 and TGF-β1↓ Foxp3 and CTLA-4↓	CD4⁺CD25^−^T cell proliferation↑ The ratio of IL-4 to IFN-γ↓	7-ds survival (2 hs before CLP) ↑
Gao, et al. (50)	Mouse	CLP	Nrp-1 (siRNA, Nrp-1 Ab)	Immediately after CLP	24 hs after CLP	Spleens and renal	Stability and activity (Foxp3, CTLA-4, TGF-β1^m+^, IL-10, and TGF-β1)↓	IL-10, IL-4, and TGF-β1↓ IFN-γ↑	Renal injury↓
Lou, et al. (77)	Mouse	CLP	LAG-3 (KO, LAG-3 Ab)	Immediately after CLP	24 hs after CLP	Blood Spleens	Percentage and absolute number↓	Cytokines (TNF-α, IL-6, and IL-10) and T cells apoptosis↓ IFN-γ, the absolute number and proliferative ability of CD4/8^+^T cells↑	7-ds survival↑ Bacterial clearance↑
Xu, et al. (82)	Mouse	CLP	CXCL4 (CXCL4 Ab)	Immediately after CLP	72 hs after CLP	Urine Blood Spleens	Percentage and absolute number↓	IL-6, IL-10and TNF-α↓	Urine creatinine and urea nitrogen↓
Gao, et al. (48)	Mouse	CLP	Sema3A (EGCG, a strong inhibitor of Sema3A)	Immediately after CLP	24 hs after CLP	Spleens Blood	Foxp3↓	IL-4↓ IFN-γ↑ Apoptosis of CD4^+^ T cells↓ Proliferative ability of CD4^+^ T cells↑	liver, lung, and renal injury↓
Brichacek, et al. (102)	Mouse	CLP	Inhibitor of TNAP (SBI-425)	Daily for 7 days after CLP	24 hs after the final injection	Plasma Brain Bone Spleens	CD4/8^+^Foxp3^+^ splenocyte T-cell populations↓	Did not affect 7-ds clinical severity outcomes Loss of barrier function in BBB endothelial cells↑ 48-hs Survival↓ Severity scores↑	
Martin et al. (51)	Mouse	CLP	Sirtuin1 (EX-527-an inhibitor)	24 hs after CLP	30 hs after CLP	Spleen	Percentage↓ CTLA-4↓	TGF-β, IL10↓ IFN-γ↑	7-ds survival↑
Gao, et al. (103)	Mouse	CLP	Tanshinone IIA		24 hs after CLP	Blood Lung Liver Renal Spleens	Percentage↓	CD3^+^CD4^+^ and CD3^+^CD8^+^ lymphocytes percentages↑, and apoptosis↓ IFN-γ and IL-2↑ IL-4 and IL-10↓ Macrophage phagocytotic activities↑	7-ds survival↑ Lung, liver, and renal injury↓ Intraperitoneal bacterial counts↓
Chen, et al. (104)	Mouse	CLP	Xuebijing injection	Immediately after CLP	36 hs after the CLP	Spleens Lung Renal	Percentage and absolute number↑ Differentiation↑ IL-10↑	Th17↓ IL-17, IL-6, and TNF-α↓	5-ds survival↑ Renal and lung injury↓
Chen, et al. (105)	Mouse	CLP	Curcumin	12 hs after CLP	Day 1, 3, 5, and 7 after CLP	Spleen Blood Renal Lung	Percentage and absolute number↑	TNF-α and IL-6↓ The proliferation of CD4+CD25− T cells↓ IL-10↑	Renal and lung injury↓ 7-ds survival↑
Xie, et al. (106)	Rats	CLP	Electroacupuncture	Immediately after CLP	48 hs after CLP	Spleens Intestinal lymph nodes	Percentage↓	TNF-α and IL-10↓ CD3^+^CD4^+^ cell↑	D-LA and DAO↓
Hou, et al. (107)	Mouse	CLP	Glutamine	2 weeks before CLP	72 h after CLP	Blood Spleens Renal	Percentage↓	Percentages of T and CD4^+^ T cells↑ IL-6 and IL-4↓ Bcl-2↑	Renal injury↓
Yeh, et al. (108)	Mouse	CLP	Arginine	1 h after CLP	12 and 24 hs after CLP	Blood Para-aortic lymph nodes Liver	Percentage↑	Percentages of CD4^+^T cells↑ Th1/Th2 ratio↑ Th17/Tregs ratio↓ IL-1β, IL-6, and TNF-α (liver) ↓	Liver injury↓
Di Caro, et al. (109)	Mouse	Injected with LPS	Dietary fiber (fiber cellulose)	2 weeks before injected with LPS	24 and 72 hs post LPS injection	Blood Liver Spleens	Suppressive function↑ Percentage (72 hs) ↑	Number and activation of splenic macrophages and DCs↓ Pro-inflammatory cytokines↓ Chemokines↓ Anergy in T cells↑ Hepatic DNA binding activity of NF-κB↓	4-ds survival↑ (110)
Albayati, et al. (111)	Mouse	CLP	P2Y12 antagonism (clopidogre)	2 hs before CLP	24 hs after CLP	Blood Spleens Hearts Renal	Percentage and absolute number (spleens)↓	Platelets and CD4^+^ T cells interactions↓	7-ds survival↑ Splenomegaly and spleen damage↓ Renal and cardiac injury↓
Sun, et al. (112)	Mouse	CLP	COX-2-specific inhibitor (parecoxib)	20 min after CLP	24 hs after CLP	Blood Spleens	Percentage↓	IgM and IgG↑ IL-1β, IL-10, and TNF-α↓	7-ds survival↑ Spleen injury↓
Ahmad, et al. (113)	Mouse	CLP	Poly (ADP-ribose) polymerase inhibitor (Olaparib)	30 min and 8 hs after CLP	24 hs after CLP	Spleens Blood	Percentage and absolute number↓	Number of CD4/8^+^ lymphocytes↑ Th17/Treg ratio↓ TNF-α, IL-1α, IL-1β, IL-2, IL-4, IL-6, and IL-12p40↓	48-hs survival (young, males) ↑ Multiorgan dysfunction↓ Bacterial CFUs↓
Cao, et al. (114)	Mouse	CLP	Ulinastatin	1 h before and 6 hs after CLP	24 hs after CLP	Spleens Blood Lung Liver Renal	Foxp3 and CTLA-4↓	Teff apoptosis↓ Teff proliferation↑ TNF-α, IL-1β, IL-2, and IL-10↓	72-hs survival ↑ Lung, liver, and renal injury↓
Topcu, et al. (115)	SD rats	CLP	Human dental follicle stem cells	Immediately or 4 hs after CLP	24 hs after CLP	Ileal tissue Spleens	Percentage↓	TNF-α↓	Ileal tissue injury↓
Chang, et al. (116)	SD rats	CLP	Adipose-derived mesenchymal stem cell-derived exosomes	3 hs after CLP	6, 16, 24, 48, and 72 hs after CLP	Blood Brain Cerebrospinal fluid	Percentage↑	TNF-α and IL-6↓ MMP-9, GFAP, F4/80 and CD14↓ L6Gy^+^/CD11^b/c+^ inflammatory cells↓ CD3^+^/CD4^+^cells↓ CD3+/CD8+ cells↓ Early/late apoptotic cells↓	Brain injury↓
Zhang, et al. (117)	Mouse	Infected with *E. coli* 0111: B4	Fresh frozen plasma	After severe sepsis (trembles, high fever, and difficulty breathing)	Recovered from endotoxemia	Blood	Differentiation and expansion↓	Galectin-9↓ The proliferation of Th1 and Th17↑ IL-1β, IL-6, and IFN-γ↑ IL-10↓	Recovered from endotoxemia↑
Kyvelidou, et al. (118)	Mouse	5 ds injection of LPS	IgG and IgM	Day 1 of LPS injection	Day 6 after LPS	Blood Spleens	CD25 and Foxp3↓	CRP↓ IL-6 and TNF-α↓ IL-18↑	7-ds survival ↑ MSS scoring↓

Compared with the control group, "↑" represents up-regulation, increase or enhancement； “↓” represents down-regulation, decrease or inhibition.

**Table 4:** Intervention studies using septic patients focused on the target of T_regs_ in sepsis.

Intervention study	Species	Model	Intervention	Intervention time	Observation time	Specimen Source	T_regs_	Immunological characteristics	Outcome
Liu, et al. (42)	Humans	Septic Patients with mechanical ventilation	Enteral nutrition	Treated within 48 hs after admission	D 1 and 7 after admission to the ICU	Blood	Percentage↑	Th17 cells and endotoxin↓	Duration of mechanical ventilation, lengths of ICU stay, hospital stay, And the incidence of ICU-AW↓
Sun, et al. (22)	Humans	Septic Patients	Enteral nutrition	Treated within 48 hs after admission	7 ds after admission	Blood	Percentage↑	Th17 percentages↓ Th17/Tregs ratios↓ IL-17, IL-23, and IL-6↓	Duration of mechanical ventilation↓ ICU stay↓
Chihara, et al. (119)	Humans	Septic shock patients with acute kidney injury	Pre-and post-dilution during continuous venovenous hemofiltration	24 hs within obtaining informed consent	6 and 24 hs after continuous venovenous hemofiltration	Blood	Induction rate↑	IL-6 and IL-10↓ Neutrophil phagocytic activity↓	No data
You, et al. (41)	Humans	Severe burn	High-volume hemofiltration	Within 3 days after burn	Days 1, 3, 5, 7, 14, 21 and 28 post-burn	Blood	Percentage↓	TNF-α, IL-1β, IL-6, IL-8, and PCT↓ HLA-DR↑	Incidence of sepsis, septic shock↓ Vasopressor↓ 90-ds survival ↑

Compared with the control group, “↑” represents up-regulation, increase or enhancement； “↓” represents down-regulation.

Over the past decade, evidence from many compelling experiments and clinical trials indicates that sepsis increases the heterogeneous characteristics of Tregs. They act on both the innate and adaptive immune systems, dampening immune functions, causing immuno-paralysis, and eventually leading to MODS and death in sepsis (14, 18, 122–125). Intervention strategies (**Tables 3** and **4**), such as human recombinant cytokines (IL-15 and IL-36) (59, 101), blocking phenotypes or chemokines [neuropilin (Nrp)-1, CTLA-4, lymphocyte activation gene (LAG)-3, and chemokine (C-X-C motif) ligand (CXCL) 4)] (50, 58, 77, 82), nutrients (glutamine) (107), inhibiting molecules [sema3A, tissue-nonspecific alkaline phosphatase (TNAP), Sirtuin1, P2Y12, COX-2, and poly ADP-ribose polymerase (PARP)] (48, 51, 102, 111–113), as well as even clinical therapeutics (high-volume hemofiltration, immunoglobulin, fresh frozen plasma, stem cells, and ulinastatin) (41, 114, 115, 117, 118) and traditional Chinese medicine (TCM) (electroacupuncture and tanshinone IIA) (103, 106), can increase the chance of survival by inhibiting the heterogeneous characteristics of Treg-induced immunosuppression. Alternatively, other studies have shown improved outcomes in sepsis by increasing the heterogeneous characteristics of Tregs to inhibit sepsis-induced SIRS through intervention strategies such as human recombinant cytokines (IL-38 and IL-7) (96, 97), blocking phenotypes or cytokines (CD28 and IL-3) (81, 95), nutrients (arginine and fiber cellulose) (108, 109), and others (bilirubin, ITK inhibitor, miR-126, maresin1, excretory-secretory products of Trichinella spiralis adult worms, and adipose-derived mesenchymal stem cell-derived exosomes) (19, 20, 70, 98, 99, 116), as well as even clinical therapeutics (enteral nutrition and pre-and post-dilution during continuous veno-venous hemofiltration) (22, 42, 119) and TCM (baicalin, rhubarb, Xuebijing injection, and curcumin) (21, 100, 104, 105). Establishment of sepsis models such as the “memory mouse” (57, 95), “two- or three-hit mouse” (70, 118), and “gene recombination mouse” models (78, 79, 94) have begun to shed light on additional heterogeneous immune characteristics in sepsis, including the presence of IL-10+ regulatory B cells (Bregs) and lipopolysaccharide-responsive beige-like anchor protein (LRBA)-deficient patients (97, 126, 127).

## Treg Checkpoints in Sepsis

Multiple co-stimulatory molecules (CD28, CD27, OX40, and 4-1BB) (128–130) and co-inhibitory receptors [B− and T−lymphocyte attenuator (BTLA), T cell immunoglobulin and mucin domain-containing-3 (TIM-3), CTLA-4, T cell immunoreceptor with immunoglobulin and ITIM domains (TIGIT), LAG-3, PD-1, and Nrp-12] (15, 50, 57, 77, 93, 131) that transmit various secondary signals play a pivotal role in the heterogeneous characteristics of Tregs and may contribute to Tregs-induced dysfunction of the whole immune system in sepsis, especially imbalanced Tregs/Tconvs (15, 74–77, 83, 132, 133). Although the innate immune system is dominant in the early stage of sepsis, Tregs are thought to be the link between the innate and adaptive immune systems (37, 40).

The percentage of Tregs, OX40+ Tregs, and 4-1BB+ Tconvs were higher in the early stage of CAP-associated septic patients. The percentage of CD4+CD27+, CD4+CD28+, and CD4+OX40+ CD27-CD28- T cells were positively correlated with SOFA and predicted 28-day mortality, respectively (40). In addition, these data indicated that imbalanced expression of OX40 and 4-1BB may contribute to evaluate the imbalance of Tregs/Tconvs. The absolute number of CD4+TIM-3+, CD4+PD-1+, and CD4+CTLA-4+ T cells were positively correlated with the severity of sepsis, especially CD4+PD-1+ T cells, which may be a risk factor for sepsis (93). BTLA is a co-inhibitory receptor that is constitutively expressed on IL-10+Tregs, which can effectively inhibit the function of CD4+ T cells (15). BTLA expression on Tregs remained high in patients with sepsis, compared to healthy controls from day 1 to 7, especially in non-survivors (75). GPR174, a member of the G-protein-coupled receptor family, plays a negative role in the development and functionality of Tregs which is highly expressed on the surface of Tregs in the early stages of sepsis and closely associated with adverse sepsis outcomes (79). A decrease of Human Leukocyte Antigen‐DR (HLA‐DR) expression on monocytes has proved to be a reliable indicator of immunosuppression in sepsis (37, 41, 60). From day 1 to 28 after sepsis diagnosis, both Foxp3 and RORC, the specific transcription factor of Tregs and Th17 cells, respectively, were significantly more highly expressed in survivors than in non-survivors. The lack of a linear correlation with HLA-DR may be due to the influence of sample size and other patient-specific factors (60). Thymus Stromal Lymphopoietin (TSLP) has been identified as a crucial inflammatory cytokine in immune homeostasis and promoted Tregs differentiation (134). The percentage of IL-10+ Tregs significantly increased in septic patients with high TSLP levels (80). A comprehensive study on the expression of co-stimulatory molecules and co-inhibitory receptors in different stages of sepsis induced Tregs would contribute to the systematic understanding of Tregs in sepsis and help people identify the most effective immune checkpoints for Tregs.

## Host-Dependent Treg Patterns in Sepsis

Compelling experimental and clinical evidence has indicated that sepsis is a multi-dimensional heterogeneous syndrome, which is reflected in the host’s variable immune responses (23, 135, 136). There is significant inter−study heterogeneity among a large number of sepsis-related studies: male sex, increased age, organ dysfunction acquired during ICU stay, recurrent sepsis, and presence of comorbidities are independently associated with increased sepsis−related mortality, especially in ICUs (2, 3, 137). Seymour and colleagues demonstrated that patients with the α phenotype (33%) have the lowest administration of a vasopressor; Patients with the β phenotype (27%) are the oldest age with the most chronic illness and renal dysfunction; Patients with the γ phenotype (27%) have the most inflammation and pulmonary dysfunction; Patients with the δ phenotype (n = 2667; 13%) have more liver dysfunction and septic shock. Their cumulative 28-day mortality rates are 5%, 24%, 13%, and 40%, respectively (135).

Studies of immune responses to sepsis usually exclude patients who have immune disorders or receive immunosuppressive medications (40, 76, 80); therefore, these studies do not fully reflect the heterogeneous characteristics of sepsis (91, 135). An increased Th17/Treg response throughout infection is most strongly associated with increased mortality among patients who are not immunocompromised; a decreased Th1/Treg response is most common among immunocompromised patients. Unexpectedly, patients who have immunocompromising comorbidities or take immunosuppressive medications do not have increased 90-day mortality, contrary to previous studies (138, 139). Immunocompromised patients with malignancies, especially those treated with chemotherapies that have adverse effects on immune function, have broadened the types and risks of drug-resistant multi-pathogenic infections (140). For example, systemic infection with *Candida albicans* (candidiasis) in tumor-bearing mice does not significantly increase the percentage of Tregs compared to the tumor group, but it significantly increases the proportion of Tregs in the spleen of the non-tumor bearing mouse. Surprisingly, systemic infection with *C. albicans* promotes the rapid growth of tumors, and the percentage of tumor-infiltrated Tregs in the tumor/candidiasis group is significantly higher than these in the tumor only group (64). This demonstrates that candidiasis could promote the growth of tumors by expanding Tregs: tumors and candidiasis promote each other through increased Treg activity. On the other hand, research on common variable immunodeficiency (CVID) and autoimmune diseases, both of which are characterized by loss of Treg function, show that the heterogeneity in sepsis due to host factors has become more prominent (127, 141). Autoimmune diseases are associated with a lower risk of 30-day death (27% reduction) for sepsis through a mechanism unrelated to the chronic immunomodulation medications (141). LRBA deficiency leads to different types of congenital immune deficiencies, such as CVID, autoimmune lymphoproliferative syndrome (ALPS) with recurrent infections, and even sepsis. Low expression of CTLA-4, Foxp3, and CD25 in LRBA-deficient patients leads to a partial loss of the regulatory effects of Tregs on T/B cell activation and causes an inappropriate increase in T and B cell activation (127).

Some evidence demonstrates that ICU-acquired infections contribute to the overall mortality of septic patients. Patients with septic shock who have secondary infections are at a 5.8 times higher risk of late-stage death than those without because of their unique immunosuppressive status, especially T cell exhaustion caused by aging and recurrent sepsis (2, 23, 52, 59, 142). In a clinically relevant cecal slurry (CS) induced model of recurrent sepsis, increased T cell exhaustion and poor prognosis (including reduced survival rate and body weight) was observed in aged (18-24 months old) compared with young (5 week old) female or male C57BL6/J mice. Their symptoms persisted for over 50 days and were associated with increased PD-1 expression on Tregs (59). Olaparib, a competitive PARP inhibitor used in the field of oncology that inhibits the binding of NAD+ to the catalytic sites of PARP, showed significant protective effects on cecal ligation perforation (CLP)-induced sepsis in young (8 weeks old) male adult mice compared with aged (72 week old) female mice (143). These age-and sex-selective protective models were associated with olaparib reducing the Treg and Th17 populations, and the Th17/Tregs ratio, by regulating intracellular miRNA levels (113).

In infants, especially preterm infants, early-onset sepsis (EOS) increases the risk of death or neurodevelopmental disorders (144). In a multi-centric clinical study of 326 neonatal intensive care units, 0.8% of infants suffered from EOS, where many factors reduced lymphocyte activation and the percentage of Tregs, including low Apgar score, caesarean delivery, small gestational age, prenatal antibiotic exposure, vitamin D deficiency, and positive maternal group B streptococcus screening results (145, 146). Intraperitoneal injection of *Escherichia coli* O55: B5 LPS in neonatal mice reduced survival and growth rates, including lung development, in a dose-dependent manner. These effects were associated with decreases in the percentage of anti-inflammatory CD4+TCRβ+Foxp3+ Tregs (86).

In addition, multiple clinical studies show that amplification of CD4+CD25+ Tregs and increased Foxp3 levels may increase risks of nosocomial infections or secondary infections in sepsis (61, 68, 147). Using a “two-hit” CLP model with intratracheal injection of *Pseudomonas aeruginosa*, which mimics clinical conditions of secondary infection, Hu *et al.* demonstrated that the absolute number of Foxp3+ Tregs in both spleen and lungs increased 24 hours after secondary *P. aeruginosa* infection. After injection of PC61 (depletion of Tregs *via* CD25), the absolute numbers of Tregs in the spleens and lungs of septic mice were reduced by 50% and 60%, respectively. Partial Treg depletion increased IL-17A, IL-1β, and IL-6 secretion, and decreased IL-10 secretion, in septic mice infected with *P. aeruginosa*, thereby reducing the bacterial load and lung injury, and improving 7-day survival (90). On the other hand, 8-week-old male C57 mice with simulated repeat infection by repeated subcutaneous injection of LPS were able to resist CLP-induced sepsis and hyper inflammatory response. These mice had an increased absolute number of Tregs and Th17 and decreased ratio of Th17/Tregs (88). However, ICU studies with critically ill lymphocytopenia patients suggested that the first three days of septic shock may be characterized by a skewed distribution of circulating innate lymphoid cells (ILC), with an excess of ILC1 and a lack of ILC3. At the same time, there was a significant decrease in the absolute number of circulating Tregs (37). These conflicting studies in both mice and humans highlight the heterogeneous nature of Tregs in sepsis that vary upon host conditions.

## Tissue-Specific Treg Patterns in Sepsis

In addition to the role Tregs play in maintaining immune homeostasis in dedicated lymphoid tissues, these cells exist in other tissues such as the lung, liver, renal, muscle, brain and myocardium (73, 87, 98, 99, 102, 148). Many tissue-specific Treg functions go beyond our initial understanding of Tregs as immune inflammation-specific inhibitors (70, 99, 133). However, most previous interventional and observational studies on sepsis have focused on the functions and characteristics of Tregs in the peripheral circulation and spleen (41, 48, 76, 96, 101). Splenectomy improved 28- day survival in a secondary sepsis CLP mouse model from 62% to 92%, which was concurrent with the lower release of inflammatory cytokines (IL-6, CXCL-1, and MCP-1) and a 41% increase in Tregs within 48 hours (65). This indicates that induced circulating Tregs (iTregs), rather than natural Tregs (nTregs) originating in the spleen, may play a role in improving sepsis survival. Sepsis has tissue-specific pathophysiological characteristics due to anatomical and histological constraints: the structure, morphology, and composition of the vasculature system vary across different organs (149–151). In mice infected with *Pseudomonas aeruginosa*, the absolute number of Foxp3+ Tregs in lung tissue increased nearly 2-fold on the third day, then gradually decreased and returned to normal on the seventh day. However, the absolute number of Foxp3+ Tregs in the spleen increased 1.6-fold on the third day and continued to increase (90).

Acute lung injury (ALI) or acute respiratory distress syndrome (ARDS) is a type of respiratory failure caused by trauma, infection (sepsis), or intoxication (152, 153). The pathophysiological mechanism of ALI/ARDS is characterized by rapid onset of widespread lung inflammation (87, 135, 154). A growing body of evidence shows that CD4+CD25+Foxp3+Tregs play a positive role in alleviating sepsis-induced rapid onset inflammation and improving the outcome of ALI/ARDS through both TGF-β-dependent and independent pathways (20, 22, 42, 81, 87, 98, 155). In a mouse model of sepsis-induced ALI, blocking HMGB1 or myeloid-specific PTEN KO (PTEN M-KO) increased TGF-β production, inhibited Rorγt and IL-17 expression, and promoted the β-catenin signalling pathway. The increased CD4+CD25+Foxp3+ Tregs in the lungs improved survival and weight outcomes. However, the opposite result was obtained with myeloid-specific β-catenin ablation (β-catenin M-KO). Furthermore, *in vitro*, the destruction of macrophage HMGB1/PTEN or activation of β-catenin significantly increased CD4+CD25+Foxp3+ Tregs (87). This also suggests that infiltration of macrophages could inhibit lung tissue-specific CD4+CD25+Foxp3+ Tregs *via* HMGB1/PTEN/β-catenin axis in sepsis-induced ALI.

The pathophysiology of sepsis-associated encephalopathy (SAE) is complex, multifactorial, and tissue-specific. Combining intertwined processes, SAE is promoted by countless alterations and dysfunctions resulting from the early and late stages of sepsis. Additionally, some patients experience chronic “sepsis brain” after sepsis recovery, such as inflammation, neuro-inflammation, oxidative stress, reduced brain metabolism, and injuries to the integrity of the blood brain barrier (BBB) (84, 156, 157). In the early stage of sepsis, some corresponding interventions are effective in alleviating the uncontrolled hyper-inflammatory (IL-1β, IL-6 IL-18, and TNF-α, etc.) and immune (CD3+CD4+ cells and CD3+CD8+ cells, etc.) responses associated with altering the BBB and amplifying the inflammatory responses of SAE (116, 156–158). Mesenchymal Stem Cell (MSC)-derived exosomes significantly increased the percentage and absolute number of Tregs, which ameliorated brain injury in the early stage of sepsis by mitigating the hyper-inflammatory and immune responses (116). As sepsis management techniques continue to improve, SAE is characterized as chronic “sepsis brain”, which is associated with long-lasting cognitive deficits and psychological impairments such as anxiety and depression (159, 160). Using a CS-induced sepsis mouse model and focusing on chronic “sepsis brain”, Saito *et al.* demonstrated that infiltrated Tregs and Th2 cells attenuate SAE and alleviate SAE-induced mental disorders by resolving neuroinflammation in the chronic phase of sepsis (84).

## Pathogen-Specific Treg Patterns in Sepsis

Many previous induced sepsis models focused on Gram-negative bacteria and their products, such as LPS (50, 51, 101, 103, 118, 121, 161). A recent experimental LPS-induced endotoxemia study in humans showed that pro-inflammatory Th1 (IFN-γ, IL-2, and TNFα) and Th17 (IL-17A) cells were suppressed, while the Tregs and their ability to produce anti-inflammatory IL-10 were not affected (162). In addition, glycolipids and diacylglycerols from *Streptococcus pneumoniae*, which cause high mortality in patients over 65-years-old, induced septic shock by activating invariant natural killer T cells (iNKT) and the hyper-inflammatory responses (66, 110, 163, 164). Tregs reduced the proliferation of iNKT and IL-4 secretion of iNKT induced by glycolipids (including bacterial-derived diacylglycerols). One striking observation was that Tregs significantly increased Foxp3 expression, inhibitory function, and IL-10 secretion after they contacted iNKT, especially in the presence of bacterial diacylglycerols (164). Recent evidence suggests that *Streptococcus pneumoniae* (including its components and live attenuated mutants) and pneumococcal infection may induce Treg proliferation and may be used in the treatment of asthma (165).

Graphene oxide (GO) is a single-atomic layered material composed of carbon with a variety of biomedical applications, such as gene delivery, stem cell differentiation, and cancer therapy (166). In addition, GO has been shown to be able to regulate innate and adaptive immune functions (92, 166, 167). *In vivo*, the administration of GO significantly improved diacylglycerols-induced septic shock and inhibited the capacity of diacylglycerols to induce iNKT-mediated trans-activation and cytokine production of innate and innate-like cells (such as dendritic cells, macrophages, and γδ T cells), which were associated with the ability to increase the amount of Foxp3+ Tregs *via* TGF-β (166). This shows that gut microbiota not only influences the gastrointestinal tract, but also supports immune cells in distal organ sites (168). In another example, dietary supplementation with nonfermentable fiber or high fiber (HF) cellulose altered the gut microbiota and positively impacted metabolic health to confer protection in sepsis models (109, 169). Supplementation with HF amplified the suppressive function of CD4+CD25+Foxp3+ Tregs, inhibited SIRS, and induced anergy in CD4+ T cells as compared to mice on a regular diet (109). These pieces of evidence also suggest that manipulating intestinal microbiota through dietary supplementation with fiber may have broader systemic effects on immune homeostasis by influencing the heterogeneity of CD4+CD25+Foxp3+ Tregs.

Fungi are involved in 20% of sepsis and Candida is the most commonly isolated pathogen (170, 171). Patients with malignancies and immunodeficiencies are more likely to develop *Candida albicans* infection that leads to candidiasis (171). *C. albicans* induces the production of tumor infiltrating and IL-10 producing Tregs through toll-like receptor (TLR) 2, which leads to immune escape (64, 172). Different degrees (such as 1,3-β-D-glucan -positive colonization and invasive candidiasis) of Candida have different effects on patients with abdominal sepsis. Decreased B and NK cell counts, and reduced IL-8 secretion appeared to be associated with a higher risk of subsequent candidiasis, rather than the heterogeneous characteristics of Tregs. In contrast, the risk stratification of candidiasis did not affect the heterogeneous characteristics of Tregs in patients with abdominal sepsis (173).

## Time-Dependent Treg Patterns in Sepsis

Considering the various failures of clinical trials targeting hyper-inflammatory mediators (especially IL-1β and TNF-α) and the fact that most septic patients who survive the acute stage of hyper-immune and inflammatory responses are burdened by secondary infections, it is necessary to perform basic and translational studies to understand the long-term post-sepsis immune perturbations (26, 27, 45, 46). The heterogeneous characteristics of Tregs are constantly changing over the course of sepsis. In the early stage of sepsis there is no difference in the percentage of Tregs in total CD4+ T cells between future sepsis survivors and non-survivors. However, non-survivors had a lower absolute number of Tregs compared to survivors. At the later stage of sepsis (after 3 days), the absolute number of Tregs increased, while the percentage of Tregs decreased in survivors. Although the absolute number of Tregs increased, the percentage of Tregs progressively increased in non-survivors. Moreover, survivors had a lower percentage of Tregs and a higher absolute number of Tregs (69, 75, 138). During the early stage of sepsis, especially with organs injuries caused by hyper-inflammatory responses (such as ALI, AKI, ALF, etc.), increasing the proportion and absolute number of Tregs is critical to restore immune-inflammatory homeostasis, and reduce tissue damage and organs injury. Animals depleted of Tregs at this stage are unable to resolve SIRS and die from extensive tissue damage and MODS (20, 21, 70, 81, 98, 104, 108, 174).

Evidence from gene knock-out (KO) mice with sepsis induced by LPS or CLP illustrates that Tregs play a crucial role in inhibiting SIRS and ameliorating acute organs injury in the early stage of sepsis (77–79, 87, 94). Gpr174 deficiency in Tregs promoted the expression of CTLA-4 and the secretion of IL-10 in CD4+CD25+Foxp3+Tregs but the expression and percentage of PD-1 and Foxp3 was not affected. In Gpr174-KO mice induced by LPS or CLP to simulate sepsis, the induction of M2 macrophages in the early stage was Treg dependent and Gpr174-deficient Tregs protected mice from sepsis-induced ALI and improved survival by promoting M2 macrophage polarization (79).

The peritoneal contamination and infection (PCI) mouse model, which is consistent with secondary infections in post-septic patients, induced an increase in Bregs but did not induce a lasting increase in Treg absolute number in the spleens from 1 week to 3.5 months after sepsis induction (97). Since the absolute number of Foxp3+ Tregs in the lung tissues of CLP-induced septic mice increased nearly two-fold on the third day and returned to normal levels on the seventh day, mice were susceptible to intratracheal injection of *Pseudomonas aeruginosa* for 3 days, but not for 7 days (90). This suggests that Tregs have different functions at different stages of sepsis and contribute to secondary *P. aeruginosa* infection. In a study with Xuebijing Injection, which contains 5 Chinese medicine herbal extracts, mice were injected once/day for 5 days after CLP. Septic mice had significantly improved 7-day survival and reduced acute organ injury, which is associated with stimulated IL-10+ Foxp3+ Tregs, inhibited Th17 differentiation, and decreased Th17/Tregs (104). Some TCM, such as rhubarb, have a bidirectional regulatory effect on the heterogeneity of Tregs over time and improve the prognosis of sepsis by increasing the proportion of Tregs in the early stage and decreasing it in the late stage, although the specific molecular mechanism of their effect is not clear (100). Although these results are contradictory, they do imply that Foxp3+ Tregs play an important role in amending early, late, and even long-term immune disturbances after sepsis.

## Limitations of Treg Models in Sepsis

While most of the data discussed in this review comes from animal models, their limitations must be acknowledged. Most previous experiments related to sepsis were induced by CLP or LPS, where researchers used inbred mice under a specific pathogen-free (SPF) experimental environment. These methods do not fully conform to clinical heterogeneity and often do not inform the treatment of sepsis in humans (50, 51, 101, 103, 118, 121, 161, 175). In fact, changes in the heterogeneity of Tregs in induced sepsis animal models do not fully reflect clinical sepsis or are even opposite to patients’ results (51, 75). Although LPS induction is a frequently used sepsis model, mice and other rodents are much less sensitive to LPS than humans. Thus, a 106 times higher (1-25 mg/kg) dose is required for mice compared to humans, who only need 2-4 ng/kg to induce SIRS (124, 162, 176). Furthermore, in most current experiments using LPS, the regimens and dosages of LPS vary widely among different mouse strains, animal ages, and animal facilities (23, 70, 87, 98, 109, 117, 118). For example, BALB/c mice induced by intraperitoneal injection of LPS (0.2 μg/g of body weight or 5 μg/mouse per day) for 5 consecutive days, showed significant decreases of CD4+, CD8+, CD3z+, and CD19+ cells and an increase of the percentage of CD25+Foxp3+ Tregs, accompanied with increased production of IL-6, TNF-α, and IL-18 in the serum. These results are consistent with the co-existence of SIRS and CARS observed in the early stage of septic patients (4, 15, 26, 45–51, 118). In a cross-design placebo-controlled study of 20 healthy male volunteers who received intravenous LPS (0.8 ng/kg body weight), their circulating neutrophils significantly increased. Additionally, the absolute numbers of CD3+, CD4+, and CD8+ T cells decreased 2 hours after LPS injection. In contrast, the frequency of Tregs and their ability to produce IL-10 did not change (162).

In the CLP model, the cecum of immunocompetent mice is sutured and then punctured to cause spillage of cecal contents into the peritoneum, which creates a life-threatening infection characterized by physical disorders (such as septic shock and acute organ failure) and ultimately death (99, 106, 161). Unfortunately, the precise composition of cecal contents that participates in the infection process is variable and has not been adequately evaluated in the case of acute organ failure (175). To compensate, some investigators tried to adopt intraperitoneal injection of stool suspension or CS, or endotracheal injection of a predetermined pathogen (such as *Klebsiella pneumonia* and *Staphylococcus aureus*, etc.) (59, 67, 97). The “two-hit” model was used to mimic clinical conditions of secondary infection, but different regimens yielded surprisingly different results (57, 70, 83, 90, 97).

Due to their relatively stable genetic uniformity, inbred BALB/c and C57BL/6 mice are most frequently used in sepsis-related studies (47, 59, 65, 70, 83, 86, 98, 118, 177). Nevertheless, researchers are beginning to emphasize the importance of using genetically heterogeneous organisms in experiments since they can better simulate the heterozygosity of humans, especially in multi-dimensional heterogeneous syndromes such as sepsis (19, 23, 67, 85, 100, 135, 136). BALB/c (inbred) and CD-1 (outbred) mice underwent unilateral femoral fracture, splenectomy, and hemorrhagic shock, with increased circulating granulocytes (LY6G+CD11+) in both strains at 24 and 48 hours later. However, CD8+ T cells decreased by 30% within 48h only in BALB/c mice. Circulating CD4+CD25+CD127low Tregs and lymphocytes (CD11B-LY6G-MHC-2+) were always at least 1.5-fold higher in BALB/c mice, while MHC-2 expression in bone marrow decreased in CD-1 mice. In addition, BALB/c mice expressed higher levels of circulatory CD4+CD25+CD127low Tregs and MHC-2+ lymphocytes, compared to CD-1 mice (178).

Based on the high heterogeneity of Tregs observed in clinical sepsis patient samples, we suggest that sepsis animal models should be designed to mimic this heterogeneity. For example, new sepsis models could be designed by guidance from both clinical sepsis patient characteristic and Treg immune checkpoints. Some experimental models of sepsis such as the “memory mouse” (57, 95), “two- or three-hit mouse” (70, 118), and “gene recombination mouse” models (78, 79, 94) have begun to move the field closer to more relevant sepsis models.

## Therapeutic Interventions Targeting Tregs

Several lines of evidence from experimental studies suggest that Tregs can be the target for therapeutic interventions. Deletion of Treg Notch4 gene with anti-Notch4 immunization in rodents normalizes dysregulated innate immunity to reduce morbidity and mortality (179). Lymphocyte-deficient recombinase activating gene-1 knockout mice exhibit impairments in lung injury healing. It has been found that administering isolated Tregs in a model of lung injury helps improve recovery (180). Depletion of Foxp3-positive Tregs from proliferating alveolar cells in a rodent model led to a decrease in epithelial proliferation (181). Such observations suggest that there are several pathways to explore regarding the therapeutic role of Tregs in sepsis. Moreover, Th17/Treg ratio alterations in favor of Th17 also have implications for therapeutic utility for lung injury and acute respiratory distress syndrome (182, 183).

## Discussion

Sepsis remains the leading cause of death in ICUs due to the progress of aging, numerous chronic comorbidities (diabetes, malignancies, autoimmune diseases, etc.), multi-drug resistant bacterial pathogens caused by excessive use of antibiotics, repeated secondary infections and other factors. The main pathologic mechanism of sepsis-induced immunosuppression is not completely understood. Furthermore, systematic, standardized clinical treatment for sepsis-induced immunosuppression is lacking. Therefore, there is an urgent need for a better understanding of the pathophysiological mechanisms of sepsis. New approaches to identify biological targets and checkpoints for detection, assessment, and management must be developed. Research that is emerging from the study of COVID-19 is likely to further inform scientists about the roles of Tregs in sepsis. In COVID-19 patients, Tregs are reported to behave variably. Whereas some studies have reported decreases in Tregs in COVID-19 patients (184, 185), others have reported increases in Tregs in COVID-19 patients (186, 187). An imbalance in the Treg/Th17 ratio in COVID-19 patients may increase the risk of respiratory failure (74, 188). Overall, to improve sepsis symptoms through the regulation of Tregs, it is necessary to find the optimal balance point for Tregs to play a role in sepsis. Researchers should not only take into account the heterogeneous characteristics of Tregs, but also the characteristics and organ/tissue-specific patterns of the host, the multi-dimensional heterogeneous syndrome of sepsis, the different types of pathogenic organisms, and even different types of laboratory research models and clinical research methods.

## Author Contributions

Conceptualization, Y-lG, YY, and Y-fC. Writing—original draft preparation, Y-lG, YY, XZ, FC, X-lM, X-sC, C-lW, and Y-cL. Writing—review and editing, Y-lG, Y-fC, and XT. Supervision, S-tS, Y-fC, and Y-lG. Funding acquisition, Y-lG and Y-fC. All authors have read and agreed to the published version of the manuscript.

## Funding

This work was supported by the National Natural Science Foundation of China (No. 81871593, 81701931), and the National Natural Science Foundation of Tianjin (No. 18JCQNJC10500).

## Conflict of Interest

XT was employed by Beijing Qiansong Technology Development Company.

The remaining authors declare that the research was conducted in the absence of any commercial or financial relationships that could be construed as a potential conflict of interest.

## Publisher’s Note

All claims expressed in this article are solely those of the authors and do not necessarily represent those of their affiliated organizations, or those of the publisher, the editors and the reviewers. Any product that may be evaluated in this article, or claim that may be made by its manufacturer, is not guaranteed or endorsed by the publisher.
